# Successful Management of Acute Puerperal Uterine Inversion After Vaginal Delivery: A Case Report of a 43-Year-Old Multiparous Woman

**DOI:** 10.7759/cureus.114509

**Published:** 2026-08-13

**Authors:** Myriem Sali, Hanane Houmaid, Basma Atik, Hamid Asmouki, Abderraouf Soumani

**Affiliations:** 1 Obstetrics and Gynecology Department, Mohammed VI University Hospital Center, Cadi Ayyad University, Marrakesh, MAR; 2 Obstetrics and Gynecology Department, Centre Hospitalier Universitaire Mohammed VI, Marrakesh, MAR

**Keywords:** anatomical stages, home delivery, life-threatening, manual reduction, obstetric emergency, postpartum hemorrhage, severe complications, surgical intervention, uterine inversion

## Abstract

Acute puerperal uterine inversion is a rare but life-threatening obstetric emergency, with a reported incidence ranging from approximately 1 in 2,000 to 1 in 20,000 deliveries. It is characterized by inward displacement of the uterine fundus into or through the endometrial cavity following childbirth. This report highlights a case of acute uterine inversion in a 43-year-old multiparous woman who developed severe postpartum hemorrhage after vaginal delivery. Physical examination revealed a protruding mass through the cervix, consistent with complete uterine inversion. Following prompt fluid resuscitation and administration of uterine relaxants, manual replacement using the Johnson maneuver was successfully performed, followed by uterotonic therapy. The patient recovered without complications and was discharged on postpartum day 4.

This case aims to increase awareness that early diagnosis and immediate uterine replacement are crucial for achieving favorable maternal outcomes and preserving reproductive potential.

## Introduction

Uterine inversion is a rare obstetric complication characterized by the inward displacement of the uterine fundus through the endometrial cavity, resulting in a “finger-glove” appearance. Although uncommon, it represents a severe obstetric emergency that may rapidly lead to massive postpartum hemorrhage and maternal hemodynamic instability. The reported incidence varies widely in the literature, ranging from approximately 1 case per 2,000 to 1 case per 20,000 deliveries [1].

The prognosis of acute uterine inversion is strongly dependent on the promptness of recognition and initiation of appropriate management. Delayed diagnosis and treatment may result in severe hemorrhage, hypovolemic shock, disseminated intravascular coagulation, and, in rare cases, maternal death [1,2]. Therefore, uterine inversion should be considered in the differential diagnosis of postpartum hemorrhage, particularly when excessive bleeding is associated with an abnormal uterine fundal contour or the presence of a vaginal mass [2].

Management requires a multidisciplinary approach involving prompt resuscitation, immediate correction of the uterine inversion, and subsequent measures to prevent recurrent uterine atony. Manual replacement of the uterus remains the first-line treatment, with surgical intervention reserved for cases in which conservative measures fail [3].

In this report, we present a case of acute puerperal uterine inversion diagnosed three hours after a home vaginal delivery in a multiparous woman. This case highlights the importance of rapid clinical recognition, timely uterine replacement, and coordinated emergency management in achieving favorable maternal outcomes.

## Case presentation

Written informed consent for publication of the case details and accompanying images was obtained from the patient. All patient information was anonymized to protect confidentiality.

A 43-year-old woman with no significant medical history was gravida 7, para 7, with seven previous uncomplicated vaginal deliveries. Because the current delivery occurred at home and the newborn remained at home after delivery, reliable information regarding gestational age, neonatal birth weight, duration of labor, and the exact interval between delivery of the baby and placenta was unavailable. The patient reported that excessive traction had been applied to the umbilical cord during the third stage of labor.

**Figure 1:**
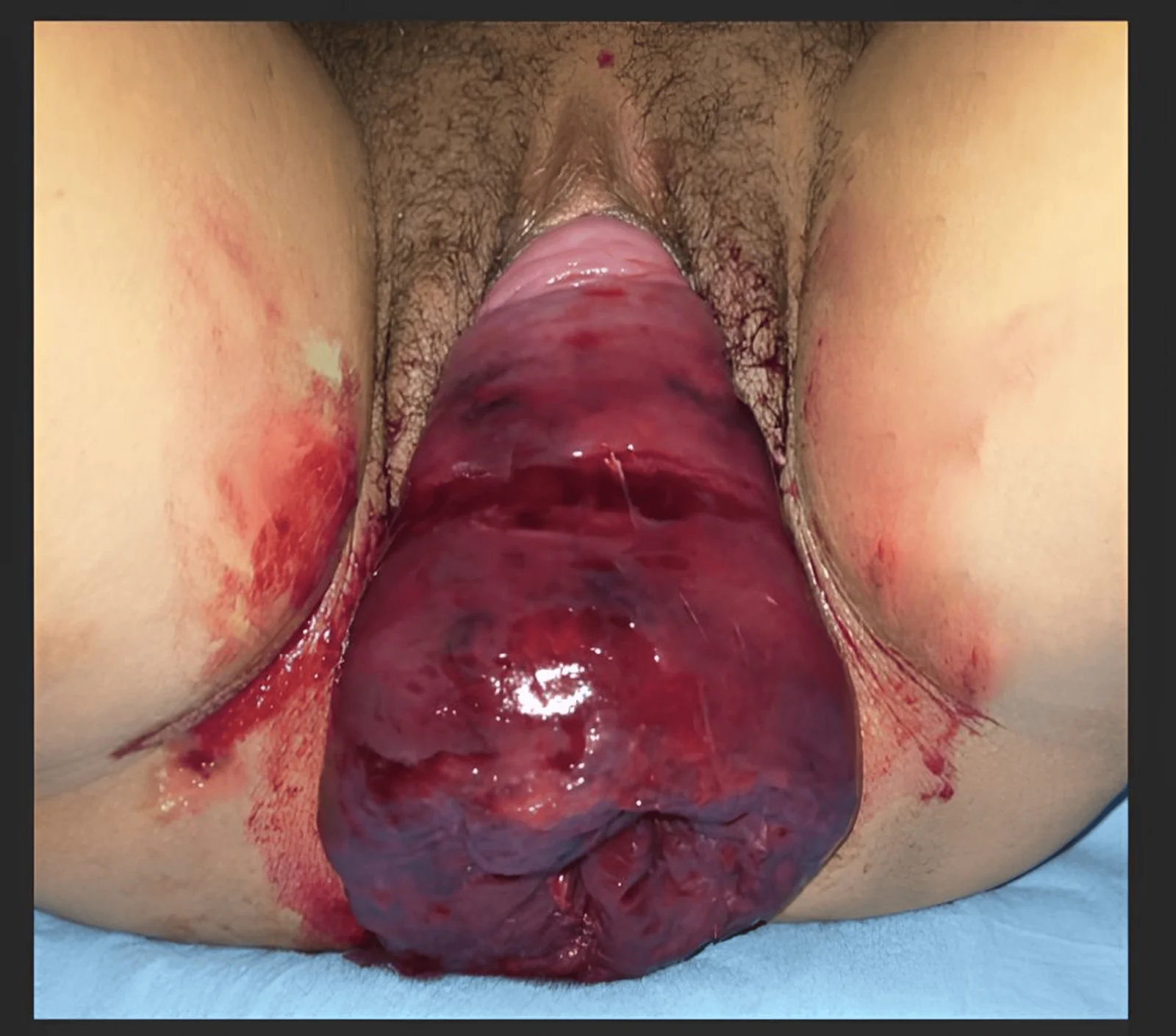
Acute uterine inversion occurring three hours after vaginal delivery

Immediately after delivery, the patient developed acute pelvic pain and postpartum vaginal bleeding. She presented to our institution three hours after the home vaginal delivery. On admission, she was conscious, with a blood pressure of 90/60 mmHg and a heart rate of 115 beats/min. Her hemoglobin level was 9.6 g/dL, and the estimated blood loss was approximately 500 mL. Initial resuscitation included placement of two large-bore intravenous lines and administration of intravenous crystalloid fluids, followed by transfusion of one unit of blood. Although she presented with borderline hypotension and tachycardia, no clinical episode of hypovolemic shock was documented. Obstetric examination revealed a large, reddish, soft, and painful mass protruding through the vaginal introitus, while the placenta had already been expelled (Figure 1). Based on the clinical findings, a diagnosis of complete acute puerperal uterine inversion (stage IV uterine inversion) was established. The patient was immediately transferred to the operating room for definitive management.

**Figure 2:**
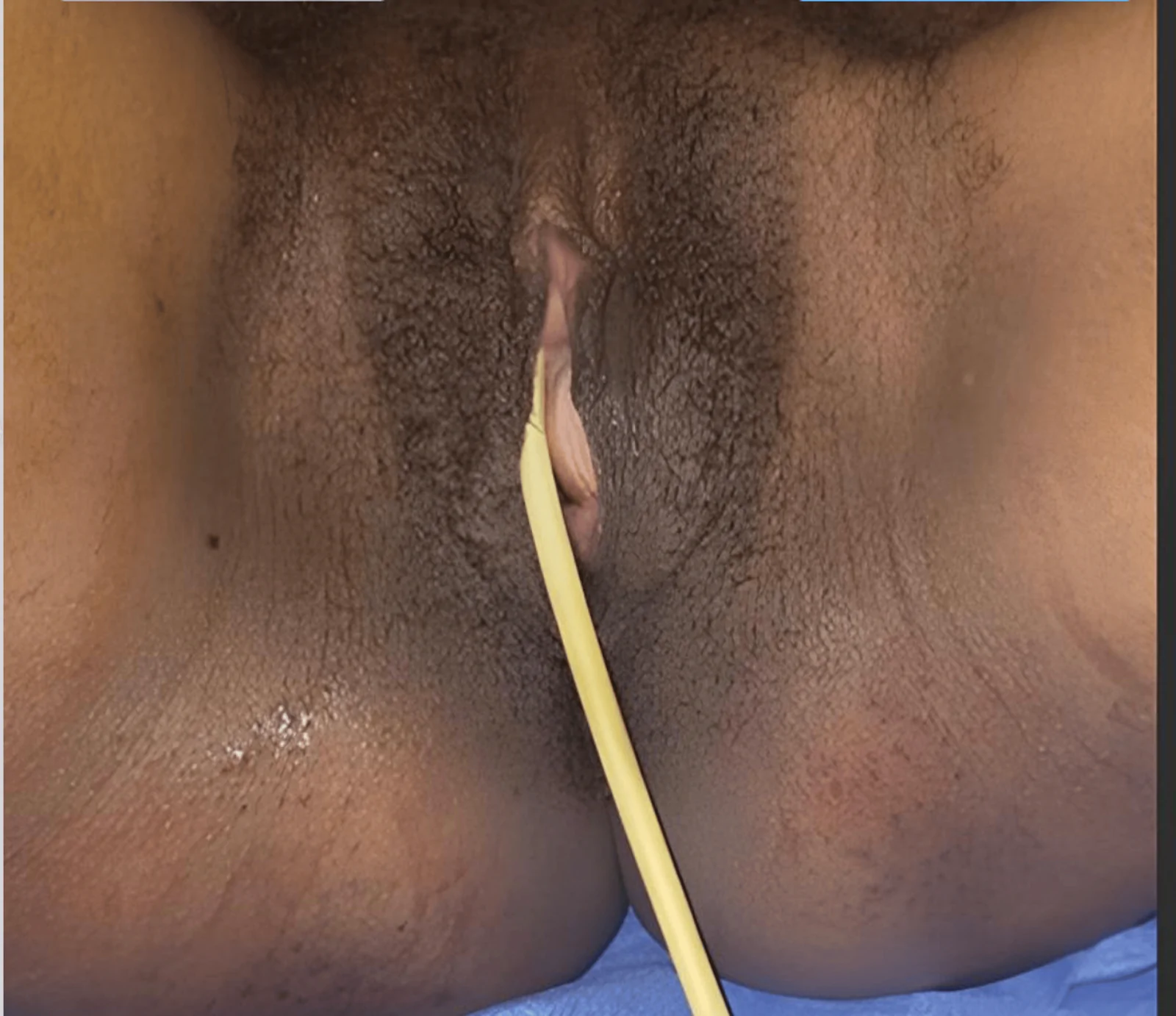
Manual reduction of acute uterine inversion using the Johnson maneuver

The patient was immediately taken to the operating room, where spinal anesthesia was administered. Adequate anesthesia and uterine relaxation were achieved, allowing successful manual reduction of the inverted uterus using the Johnson maneuver. The uterine fundus was progressively elevated with the operator’s hand, applying gentle upward pressure along the axis of the vagina while avoiding excessive force to minimize the risk of uterine injury. Successful restoration of the normal uterine anatomy was immediately followed by the administration of uterotonic agents and continuous uterine massage for approximately 20 minutes to promote adequate uterine contraction and reduce the risk of recurrence (Figure 2).

Postoperatively, the patient received prophylactic antibiotic therapy. Histopathological examination of the placenta revealed no abnormal findings. The postoperative course was uneventful, with satisfactory uterine involution and effective uterine contractions. The patient was discharged on postpartum day 4 in stable condition, with a hemoglobin level of 9.6 g/dL, compared with 11.8 g/dL before delivery (reference range: 12-16 g/dL).

At the six-week postpartum follow-up visit, pelvic ultrasonography demonstrated favorable uterine recovery. The patient reported no abnormal vaginal bleeding or other complications.

Clinical timeline

Immediately after delivery, the patient developed acute pelvic pain and vaginal bleeding following the home vaginal delivery. Three hours postpartum, she presented to our institution, where complete acute puerperal uterine inversion was diagnosed clinically. On admission, her blood pressure was 90/60 mmHg, heart rate was 115 beats/min, hemoglobin level was 9.6 g/dL, and estimated blood loss was approximately 500 mL. Initial resuscitation was performed, including intravenous fluid administration and transfusion of one unit of blood. Immediately after diagnosis, the patient was transferred to the operating room. Under spinal anesthesia, successful manual uterine reduction was achieved using the Johnson maneuver, followed by administration of uterotonic agents and continuous uterine massage. The postoperative course was uneventful, and the patient was discharged in stable condition on postpartum day 4. At the six-week postpartum follow-up visit, she demonstrated favorable clinical recovery without reported complications.

## Discussion

Acute puerperal uterine inversion is an uncommon but potentially fatal obstetric emergency. Its incidence is estimated to range from approximately 1 in 2,000 to 1 in 20,000 deliveries, although the exact frequency varies among studies because of its rarity and differences in reporting methods [1-5]. Despite its low incidence, this condition requires immediate recognition and management because maternal morbidity is primarily associated with severe postpartum hemorrhage and the rapid progression to hypovolemic shock [1,2].

Our case shares several features with previously reported cases of puerperal uterine inversion but also illustrates the particular challenges associated with home delivery. Previous reports have described uterine inversion following home delivery, with presentations ranging from acute cases diagnosed shortly after delivery to delayed presentations requiring surgical management [6-10].

In our patient, the inversion occurred in the context of excessive umbilical cord traction during the third stage of labor, and the diagnosis was established three hours after delivery based on the characteristic finding of a painful mass protruding through the vaginal introitus. Despite vaginal bleeding and borderline hypotension with tachycardia on admission, no clinical signs of shock were documented. Immediate transfer to the operating room allowed successful manual reduction using the Johnson maneuver without the need for laparotomy or hysterectomy. This favorable outcome emphasizes the importance of early recognition and prompt uterine replacement. The home-delivery setting also highlights the importance of appropriate management of the third stage of labor, avoidance of excessive cord traction, early recognition of uterine inversion, and rapid referral to an appropriately equipped obstetric facility [10].

Several factors have been associated with an increased risk of uterine inversion. The most frequently reported include excessive traction on the umbilical cord before complete placental separation, excessive fundal pressure, multiparity, and rapid or unattended labor. Additional contributing factors include uterine overdistension, abnormal placental implantation, manual removal of the placenta, a short umbilical cord, fetal macrosomia, and previous uterine inversion [2-7]. In the present case, the delivery occurred at the patient's home, as reported by the patient on admission. Excessive traction on the umbilical cord during the third stage of labor was identified from her history as a possible precipitating factor. The patient was multiparous (G7P7), representing another recognized risk factor. The exact duration of the third stage of labor could not be established because the delivery occurred outside the hospital and no contemporaneous obstetric records were available. Other potential risk factors could not be reliably assessed because of the limited obstetric information available following the home delivery.

The clinical diagnosis of acute uterine inversion is primarily based on physical examination. Typical clinical features include postpartum hemorrhage, pelvic or abdominal pain, maternal hemodynamic instability, and visualization or palpation of a mass protruding through the vagina [3-8]. Because postpartum hemorrhage has multiple possible etiologies, including uterine atony and retained placental tissue, careful vaginal examination is essential when uterine inversion is suspected. Uterine inversion should be considered whenever hemorrhage is associated with the absence of a palpable uterine fundus on abdominal examination or the presence of a vaginal mass [2-9].

Four degrees of uterine inversion have been described according to the extent of uterine displacement: Stage I, indentation of the uterine fundus without passage through the cervical os; Stage II, the inverted fundus extends through the cervical os; Stage III, the uterine fundus reaches or protrudes beyond the vaginal introitus; and Stage IV, inversion involves the uterine corpus, cervix, and vaginal walls.

Acute uterine inversion generally occurs immediately after delivery, most commonly within the first 30 minutes postpartum. When diagnosis and reduction are delayed, cervical edema and the formation of a constriction ring may develop, making uterine replacement more difficult. Inversions diagnosed beyond the acute period are classified as subacute or chronic, depending on the time interval after delivery [2].

Management relies on simultaneous resuscitation and rapid correction of the anatomical defect. Initial treatment includes stabilization of the patient with intravenous fluids and blood products, when required, followed by immediate attempts at manual uterine replacement. Early reduction is essential because prolonged inversion increases the risk of hemorrhage, infection, and uterine tissue compromise [3-9]. Adequate uterine relaxation facilitates successful repositioning and may be achieved using general or regional anesthesia or pharmacological agents such as beta-adrenergic agonists, nitroglycerin derivatives, or magnesium sulfate [3,9].

The Johnson maneuver remains the first-line technique for manual reduction. It consists of gently pushing the inverted uterine fundus upward through the vagina until normal uterine anatomy is restored. When manual replacement is unsuccessful, alternative approaches may be considered. Hydrostatic reduction involves the instillation of warm sterile fluid to generate vaginal pressure and facilitate uterine repositioning. Surgical procedures, including the Huntington and Haultain techniques, are reserved for refractory cases. The Huntington procedure involves abdominal access with progressive traction on the round ligaments, whereas the Haultain procedure requires an incision of the constriction ring to facilitate uterine replacement [9].

Following successful reduction, uterotonic agents should be administered to restore uterine tone and decrease the risk of recurrent inversion. If conservative management fails or severe complications occur, hysterectomy may occasionally be required, particularly in cases of abnormal placentation or irreducible inversion.

The outcome of uterine inversion is strongly influenced by the promptness of diagnosis and treatment. Several previously reported cases have described puerperal uterine inversion following vaginal delivery, including cases occurring after home delivery. Sena-Martins et al. reported an acute uterine inversion following home delivery complicated by hypovolemic shock; attempts at manual reduction were unsuccessful, and the patient ultimately required laparotomy, the Huntington procedure, and hysterectomy [10]. Similarly, a case reported from Morocco described a subacute uterine inversion following home delivery in a grand multiparous woman, requiring surgical management after delayed presentation [11]. In contrast, Gao et al. reported successful manual transvaginal repositioning of an acute puerperal uterine inversion, with rapid improvement in vaginal bleeding and discharge three days after delivery [12]. In our case, the diagnosis was established three hours after the home vaginal delivery based on characteristic clinical findings, and immediate manual reduction using the Johnson maneuver successfully restored the uterine anatomy without the need for laparotomy or hysterectomy. These cases highlight the variability in clinical presentation and management and emphasize that early recognition, prompt resuscitation, and timely uterine replacement are essential for achieving favorable maternal outcomes and preserving the uterus when possible [10-12].

## Conclusions

Acute puerperal uterine inversion remains a rare but potentially fatal obstetric emergency requiring immediate recognition and effective management. In the setting of severe postpartum hemorrhage, particularly when the uterine fundus cannot be palpated abdominally or a vaginal mass is identified, clinicians should promptly consider this diagnosis. Early resuscitation and rapid restoration of normal uterine anatomy are essential to control hemorrhage, prevent severe maternal complications, and preserve future reproductive potential. This case highlights that timely intervention and appropriate multidisciplinary management can lead to favorable maternal outcomes, even in cases of complete uterine inversion.
